# Changes in liver enzymes are associated with changes in insulin resistance, inflammatory biomarkers and leptin in prepubertal children with obesity

**DOI:** 10.1186/s13052-023-01434-7

**Published:** 2023-03-09

**Authors:** Rosario Valle-Martos, Luis Jiménez-Reina, Ramón Cañete, Rosario Martos, Miguel Valle, María Dolores Cañete

**Affiliations:** 1 Maimonides Biomedical Research Institute of Córdoba (IMIBIC), Córdoba, Spain; 2 Faculty of Medicine, University of Córdoba, IMIBIC, Córdoba, Spain; 3 Pozoblanco Health Centre, IMIBIC, Córdoba, Spain; 4 Valle de los Pedroches Hospital, IMIBIC, Córdoba, Spain

**Keywords:** Liver enzymes, Inflammation, Childhood obesity, Insulin resistance, Leptin, Prepubertal children

## Abstract

**Background:**

Non-alcoholic fatty liver disease is associated with obesity. A subclinical inflammation state, endothelial dysfunction, and parameters related to metabolic syndrome (MetS), have been documented in children with obesity. We aimed to determine the changes that occur in liver enzymes levels in response to the standard treatment of childhood obesity, also assessing any associations with liver enzyme levels, leptin, and markers of insulin resistance (IR), inflammation, and parameters related to MetS in prepubertal children.

**Methods:**

We carried out a longitudinal study in prepubertal children (aged 6–9 years) of both sexes with obesity; a total of 63 participants were recruited. Liver enzymes, C-reactive protein (CRP), interleukin-6, neutrophil-to-lymphocyte ratio (NLR), platelet-to-lymphocyte ratio (PLR), soluble intercellular adhesion molecule-1 (sICAM-1), leptin, homeostasis model assessment for IR (HOMA-IR), and parameters related to MetS were measured.

**Results:**

After standard treatment for 9 months, children who lowered their standardised body mass index (SDS-BMI) had significantly lower systolic blood pressure (*p* = 0.0242), diastolic blood pressure (*p* = 0.0002), HOMA-IR (*p* = 0.0061), and levels of alanine aminotransferase (ALT) (*p* = 0.0048), CRP (*p* = 0.0001), sICAM-1 (*p* = 0.0460), and IL-6 (*p* = 0.0438). There was a significant association between the changes that occur with treatment, in the ALT levels, and changes in leptin (*p* = 0.0096), inflammation biomarkers [CRP (*p* = 0.0061), IL-6 (*p* = 0.0337), NLR (*p* = 0.0458), PLR (*p* = 0.0134)], and HOMA-IR (*p* = 0.0322).

**Conclusion:**

Our results showed that a decrease in ALT levels after the standard treatment for 9 months was associated with favourable changes in IR markers (HOMA-IR) and inflammation (IL-6, CRP, NLR, and PLR).

## Background

In children with obesity, metabolic syndrome (MetS) begins at a very early age [1]. Along with the disorders that define MetS, several authors, have detected alterations indicative of endothelial dysfunction, low-grade systemic inflammation, and alterations in adipokine levels in children with obesity [2–4]. Some researchers consider non-alcoholic fatty liver disease (NAFLD) as a hepatic manifestation of MetS because it is associated with important components of this syndrome, including obesity, insulin resistance (IR), and increased triglycerides (TG) levels [5–7].

There is an association between IR, hepatic steatosis, and glucose metabolism in young people [8]. The prevalence of NAFLD increases in parallel with the prevalence of MetS and its components, particularly obesity and diabetes mellitus, and has now become the most common cause of chronic liver disease in children and adults [9–11]. The pathogenesis of NAFLD involves dietary factors, inflammation, IR, and adipocytokines, among other factors. Adipose tissue produces and releases cytokines with pro- and anti-inflammatory activity that can play key roles in the pathogenesis and progression of NAFLD by contributing to low-grade inflammation [12] and IR. Moreover, in adults, levels of serum leptin have been associated with the severity of NAFLD [13].

The liver enzyme alanine aminotransferase (ALT) is most closely related to the accumulation of fat in the liver. This enzyme has also been correlated with obesity and various components of MetS, with increased ALT being associated with a higher incidence of MetS, diabetes mellitus, and cardiovascular disease [14, 15]. Furthermore, ALT is considered a predictive factor for non-alcoholic steatosis [16] and has been associated with IR and cardiovascular risk in adolescents [17, 18]. An increase in butyryl cholinesterase (BChE) has also been observed in association with NAFLD and could represent a marker for increased fatty infiltration in the liver. In line with this, elevated levels of BChE have been described in obese individuals with MetS [19].

NAFLD is closely related to obesity, sedentary lifestyles, and high-calorie diets [10]. Of note, the high prevalence of this disease and its possible serious health consequences, make its early detection particularly important because simple steatosis is reversible through lifestyle modifications, especially weight loss [20].

The metabolic disturbances that accompany obesity, NAFLD, and MetS appear to begin in children with obesity at a very early age. In a previous cross-sectional study, our group described elevated values for liver enzymes in prepubertal children with obesity compared to children with normal weight of the same age [21]. We found association between this obesity-associated metabolic disorders and liver enzymes levels. Nonetheless, to date, very few studies in prepubertal children with obesity have analysed the effect of obesity treatments on liver enzyme levels and their impact on metabolic disorders associated with obesity and NAFLD. In our hypothesis, the standard treatment of obesity, when commenced at early stages, leads to an improvement in liver enzyme levels which is associated with favorable changes in parameters related to obesity and NAFLD. Thus, in this work we aimed to determine these changes in response to the standard treatment of childhood obesity in prepubertal children, also assessing any associations with liver enzyme levels, leptin, and markers of IR, inflammation, and parameters related to MetS.

## Materials and methods

### Study design

We carried out a longitudinal study in 63 prepubertal children with obesity (aged 6–9 years) of both sexes. Childhood obesity was defined according to Cole et al. [22] using the age- and sex-specific cut-off points of BMI corresponding to the adult cut-off of 30 kg/m^2^. We employed consecutive sampling and jointly carried out the study at the Endocrinology Section in the Reina Sofía University Hospital in Cordoba, the Clinical Analysis Service at the Valle de los Pedroches Hospital in Pozoblanco, Cordoba, and the Health Centre of Pozoblanco.

All the children in this study were Caucasian and prepubertal (Tanner stage 1). The study protocol complied with Helsinki Declaration Guidelines and was approved by the Research and Ethics Commission, North Sanitary Area of Cordoba, Valle de los Pedroches Hospital, Córdoba, Spain. All the parents of the children included in this study gave their written consent. The inclusion criteria were prepubertal age and absence of congenital metabolic diseases. The exclusion criteria were non-Caucasian, pubertal stage, children with diabetes, impaired fasting glucose, primary hyperlipidaemia, hypertension, or secondary obesity. None of the participants were receiving any regular treatments with any medications. None of the children presented fever or clinical signs of infection. Children with CRP levels > 10 mg/L (which thus indicated the presence of clinically relevant inflammatory conditions), or aspartate aminotransferase levels > 40 U/L, were excluded.

The standard treatment of child obesity consists of behavioral components, physical exercise and nutritional education, according to the recommendations of the Nutrition Committee of the Spanish Association of Paediatricians [23, 24]. It is recommended for all children with obesity who visit their pediatrician and it was also recommended in this study, without any modification. There is no random assignment and the assignment of the medical intervention is not at the discretion of the investigator, observational study. The evidence suggests that management should involve the whole family and focus on changes in sedentary behavior, physical activity, and diet.

In first visit, children who did not meet the selection criteria were excluded and the family's lifestyle, physical activity, diet, sleep habits, hours of television, and sedentary behavior were evaluated. Issues that were recognized during the initial evaluation as possibilities for improvement determined the individual priorities. None of the participants were receiving any treatments with any medications.

Changes in blood pressure, or anthropometric or biochemical parameters were monitored in all the children. At the end of the study period, we compared the children with obesity who had substantially decreased their standardised body mass index (SDS-BMI) with those whose SDS-BMI status had remained stable. A considerable decrease in SDS-BMI was defined as a reduction by 0.5 or more kg/m^2^. The remaining children were considered part of the group whose SDS-BMI had not substantially changed. We also examined the results according to the ALT expression terciles.

### Blood sampling and analysis

Blood samples were drawn after an overnight fast. ALT, AST, BChE, glucose, total cholesterol, high-density lipoprotein cholesterol (HDLc), and TG concentrations were measured using a random-access analyser (ADVIA 1800; Siemens Healthcare Diagnostics, München, Germany) with reagents from Siemens Healthcare Diagnostics. Insulin was quantified using an UniCel DxI 800 Access Immunoassay System (Beckman Coulter, Brea, Calif., USA).

Antigenic immunoassay methods were used to quantify interleukin 6 (IL-6; Quantikine human IL-6, RD systems, Wiesbaden-Norderstedt, Germany) and leptin (Quantikine human leptin, RD systems), and soluble intercellular adhesion molecule-1 (sICAM-1) was measured by ELISA (IBL Immuno-Biological Laboratories, Hamburg, Germany) using a microtitre plate analyser (Personal LAB, Phadia Spain S.L. Barcelona. Spain). C-reactive protein (CRP) was measured by nephelometry (N High Sensitivity CRP reagent, Behringwerke AG, Marburg, Germany) in a Dade Behring Analyzer II Nephelometer (Dade Behring, Inc., Deerfield, IL, USA). Finally, blood cells were counted with a haematology autoanalyzer (ADVIA 2120i Hematology System; Siemens Healthcare Diagnostics, München, Germany).

### Statistical analysis

Statistical analysis was performed using SPSS software for Windows (version 24, IBM Corp., Armonk, NY), excluding any outlying values. The results were expressed as the mean ± standard error mean (*SEM*), with a 95% confidence interval (95% CI). We tested the level of departure from the Gaussian distribution for each variable and variance equality was controlled using Snedecor’s *F*-test. The mean values of the groups were compared using Student *t*-tests. Statistical significance was set at *p* < 0.05. Correlation between the variables in the longitudinal study were evaluated using Pearson correlation coefficient and regression analyses. Multivariate regression analysis was performed using the stepwise method. For each variable, potential confounding factors (0.05 < *p* < 0.2) were evaluated by analysing the raw and adjusted regression coefficients.

## Results

### Comparison between obese children who did or did not decrease their SDS-BMI after nine months

All the variables were measured at baseline and again after standard treatment for 9 months. After 9 months, 31 children with obesity had decreased their SDS-BMI by a mean of 0.996 kg/m^2^, and while 32 children with obesity had maintained a stable SDS-BMI with an average change of 0.008 kg/m^2^. At baseline, the two groups were similar in terms of age, sex, and anthropometric measurements, and showed no differences in ALT, AST, BChE, IL-6, CRP, sICAM, the neutrophil-to-lymphocyte ratio (NLR), platelet-to-lymphocyte ratio (PLR), glucose, insulin, HOMA-IR, or leptin.

**Fig. 1:**
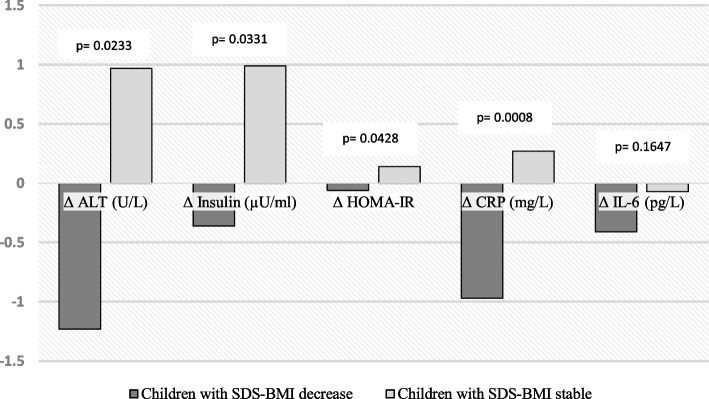
Relative changes (Δ) of alanine aminotransferase, insulin resistance and inflammatory biomarkers. Legend. Comparison of children with obesity with substantial SDS-BMI (decrease in SDS-BMI of ≥ 0.5) and children with obesity with stable SDS-BMI status. The relative changes (Δ) = the value after nine months of treatment minus the baseline values. A positive value indicates a increase, and a negative value indicated an decrease

Table 1 shows comparison of children with obesity with substantial decrease in SDS-BMI and children with obesity with a stable SDS-BMI status after nine months and relative changes of variables during intervention (Fig. 1). After standard treatment for 9 months, the mean ALT levels were significantly lower in children whose SDS-BMI had decreased, at 18.19 U/L (95% CI [16.89,19.49]) compared to 21.13 U/L (95% CI [19.54, 22.71]) in the children with a stable SDS-BMI. Compared to the group of children with a stable SDS-BMI, there was also a significant decrease in the insulin, HOMA-IR values, and expression of biomarkers for inflammation (IL-6, CRP, NLR, and PLR), endothelial dysfunction, (sICAM), leptin, and systolic and diastolic blood pressure, along with a significant increase in HDLc, in the children with obesity whose SDS-BMI had decreased (Table 1).

**Table 1:** Comparison between the two groups of children with obesity after nine months of treatment

	Children with obesity		Children with obesity		
	(SDS-BMI stable) (*n* = 32)		(SDS-BMI decrease) (*n* = 31)		
	Relative changes (Δ^a^)	Mean ± S.E.M	Relative changes (Δ^a^)	Mean ± S.E.M	p
Age (years)	0,88 ± 0,05	8.88 ± 0.17	0,87 ± 0,04	8.84 ± 0.18	0.8738
Male/Female		15/17		14/17	
ALT (U/L)	0.97 ± 0.63	21.13 ± 0.78	-1.23 ± 0.85	18.19 ± 0.63	0.0048
AST (U/L)	-0.03 ± 0.46	23.95 ± 0.71	-0.97 ± 0.50	24.32 ± 0.67	0.6986
BChE (U/L)	201.07 ± 126.38	11,027.3 ± 209.1	-880.19 ± 128.67	10,555.9 ± 226.9	0.1314
BMI (Kg/m^2^)	0.56 ± 0.13	24.19 ± 0.40	-1.40 ± 0.12	21.98 ± 0.38	0.0002
SDS-BMI	-0.047 ± 0.05	3.29 ± 0.18	0.95 ± 0.07	2.40 ± 0.19	0.0014
Waist circumference (cm)	4.04 ± 0.85	78.33 ± 1.29	-1.15 ± 0.44	71.46 ± 1.19	0.0002
Glucose (mmol/L)	-0.04 ± 0.08	5.02 ± 0.06	0.02 ± 0.07	5.07 ± 0.06	0.5680
Insulin (µU/mL)	0.99 ± 0.41	7.94 ± 0.46	-0.35 ± 0.42	5.95 ± 0.32	0.0007
HOMA-IR	0.138 ± 0.12	1.714 ± 0.10	-0.056 ± 0.14	1.347 ± 0.08	0.0061
Total cholesterol (mmol/L)	-0.05 ± 0.06	4.39 ± 0.59	-0.06 ± 0.08	4.55 ± 0.64	0.3061
Triglycerides (mmol/L)	0.09 ± 0.04	0.87 ± 0.27	0.02 ± 0.05	0.80 ± 0.18	0.2322
HDL-cholesterol (mmol/L)	0.01 ± 0.02	1.29 ± 0.14	0.08 ± 0.03	1.43 ± 0.20	0.0020
IL-6 (pg/mL)	-0,06 ± 0,15	1.64 ± 0.15	0,41 ± 0,20	1.25 ± 0.12	0.0438
CRP (mg/L)	0.27 ± 0.26	2.77 ± 0.34	-0.97 ± 0.24	1.19 ± 0.19	0.0001
NRL	0,01 ± 0,06	1.299 ± 0.09	-0,19 ± 0,06	1.069 ± 0.04	0.0286
PRL	4,01 ± 3,21	110.47 ± 4.79	-16,23 ± 3,64	97.69 ± 4.69	0.0524
Leptin (ng/mL)	6,53 ± 1,45	27.0 ± 2.10	-0,47 ± 1,42	16.93 ± 1.31	0.0002
sICAM-1 (ng/mL)	4,26 ± 14,04	281.38 ± 8.0	-21,94 ± 7,32	259.97 ± 6.77	0.0460
SBP (mm Hg)	-0,89 ± 1,41	101.78 ± 1.21	-3,08 ± 1,83	97.48 ± 1.41	0.0242
DBP (mm Hg)	-1,14 ± 1,12	62.09 ± 0.71	-4,95 ± 1,12	57.090 ± 1.06	0.0002

^a^Relative changes (Δ) the value after nine months of treatment minus the baseline values. A positive value indicates a increase, and a negative value indicated an decrease

*ALT* Alanine aminotransferase, *AST* Aspartate aminotransferase, *BChE* Butyryl cholinesterase, *BMI* Body mass index, *SDS-BMI* Body mass index standard deviation score, *HOMA-IR* Homeostasis model assessment for insulin resistance, *IL-6* Interleukin-6, *CRP* C-reactive protein, *NRL* Neutrophil-to-lymphocyte ratio, *PRL* Platelet-to-lymphocyte ratio, *sIACM-1* Soluble intercellular adhesion molecule-1, *SBP* Systolic blood pressure, *DBP* Diastolic blood pressure

### Characteristics of children with obesity stratified by alanine transaminase, divided into terciles

Table 2 shows variance analysis for all the children with obesity (*n* = 63) after treatment for 9 months, stratified by ALT expression terciles.

**Table 2:** Variance analysis in the complete group of children with obesity (*n* = 63)

	1^er^ Tercile ALT	2º Tercile ALT	3^er^ Tercile ALT	F	p
ALT (U/L)	15.48 ± 0.36	19.26 ± 0.18	24.18 ± 0.72	209.83	< 0.001
Age (years)	8.69 ± 0.24	9.10 ± 0.19	8.97 ± 0.19	1.18	0.318
BMI (Kg/m^2^)	21.17 ± 1.19	22.88 ± 0.39	24,68 ± 0.47	4.91	0.025
SDS-BMI	2.46 ± 0.31	2.71 ± 0.17	3.50 ± 0.202	4.90	0.017
WC	71.95 ± 1.48	74.51 ± 1.37	78.40 ± 1.94	3.91	0.028
Glucose (mmol/L)	5.05 ± 0.08	4.98 ± 0.09	5.11 ± 0.06	0.72	0.495
Insulin (pmol/L)	43.06 ± 3.43	47.41 ± 3.65	59.44 ± 3.72	4.17	0.023
HOMA-IR	1.275 ± 0.11	1.491 ± 0.11	1.791 ± 0.12	5.07	0.011
Leptin (ng/mL)	16.61 ± 1.74	21.37 ± 2.17	26.35 ± 2.27	5.29	0.009
AST (U/L)	23.10 ± 0.90	23.82 ± 0.48	25.26 ± 0.96	1.99	0.150
BChE (U/L)	9514.8 ± 314.4	10,026.6 ± 145.8	10,294.2 ± 299.4	2.47	0.098
SBP (mm Hg)	98.71 ± 1.59	100.86 ± 1.12	100.38 ± 1.67	0.50	0.605
DBP (mm Hg)	60.43 ± 1.46	58.67 ± 0.90	59.81 ± 1.26	0.55	0.581
TC (mmol/L)	4.46 ± 0.12	4.44 ± 0.11	4.50 ± 0.17	0.06	0.943
TG (mmol/L)	0.78 ± 0.04	0.81 ± 0.04	0.92 ± 0.06	2.01	0.147
HDL-c (mmol/L)	1.37 ± 0.05	1.35 ± 0.03	1.36 ± 0.05	0.047	0.934
CRP (nmol/L)	18.96 ± 4.48	16.48 ± 2.47	25.10 ± 3.31	1.58	0.219
IL-6 (pg/mL)	1.36 ± 0.18	1.47 ± 0.08	1.52 ± 0.21	0.21	0.784
NLR	1.26 ± 0.14	1.05 ± 0.05	1.19 ± 0.07	1.37	0.266
PLR	97.27 ± 5.97	109.59 ± 6.39	103.27 ± 5.69	1.10	0.338
sICAM-1 (ng/mL)	278.51 ± 11.61	266.64 ± 8.33	267.40 ± 7.81	0.53	0.594

Results after treatment for nine months, stratified by ALT divided into terciles

*ALT* Alanine aminotransferase, *BMI* Body mass index, *WC* Waist circumference, *HOMA-IR* homeostasis model assessment for insulin resistance, *AST* Aspartate aminotransferase, *BChE* Butyryl cholinesterase, *SBP* Systolic blood pressure, *DBP* Diastolic blood pressure, *TC* Total cholesterol, *TG* Triglycerides, *HDL-c* HDL-cholesterol, *CRP* C-reactive protein, *IL-6* Interleukin-6, *NLR* Neutrophil-to-lymphocyte ratio, *PLR* Platelet-to-lymphocyte ratio, *sICAM-1* Soluble intercellular adhesion molecule-1. Results are expressed as the mean ± S.E.M

Children in the third tercile had significantly higher SDS-BMIs (*p* = 0.017), WC (*p* = 0.028), and insulin (*p* = 0.023), HOMA-IR (*p* = 0.011), and leptin levels (*p* = 0.009). The inflammation marker values (CRP and IL-6) were also higher in the third tercile, although these differences were not significant. Blood pressure and lipid parameters were not significantly different between the terciles.

### Association between changes in alanine transaminase levels and the other variables after nine months

**Fig. 2:**
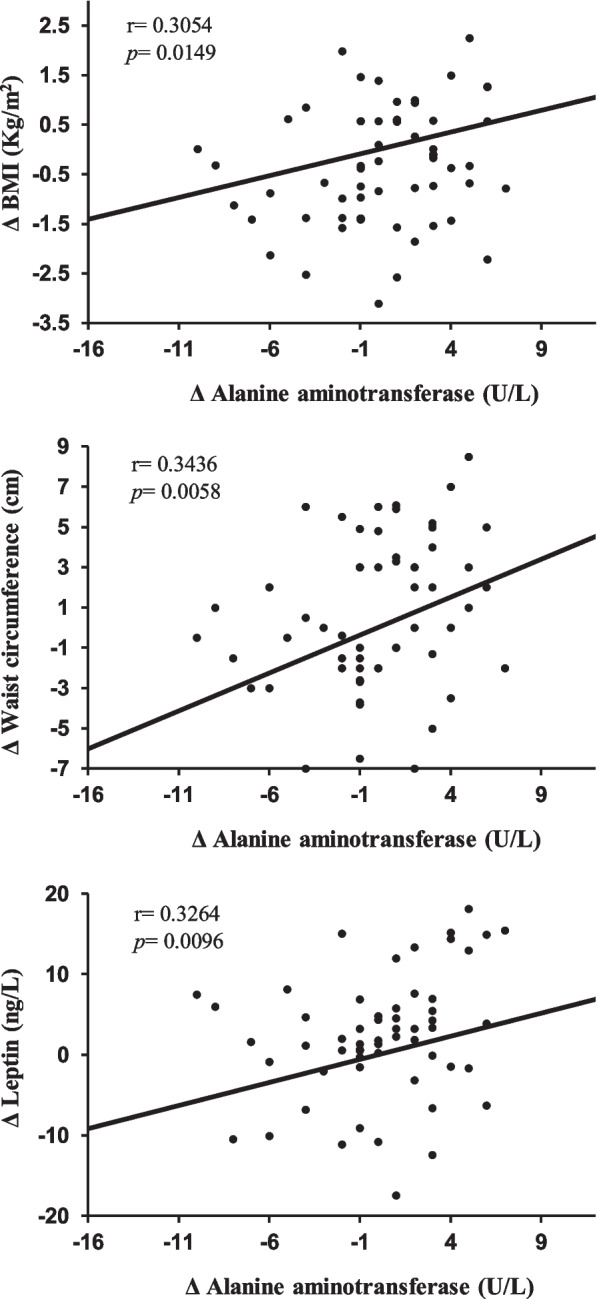
Serum Δ ALT concentrations as a function of Δ leptin, Δ WC, and Δ BMI. Legend. Changes (Δ) in alanine aminotransferase, leptin, waist circumference, and body mass index levels are expressed as values after nine months of treatment minus the baseline values

**Fig. 3:**
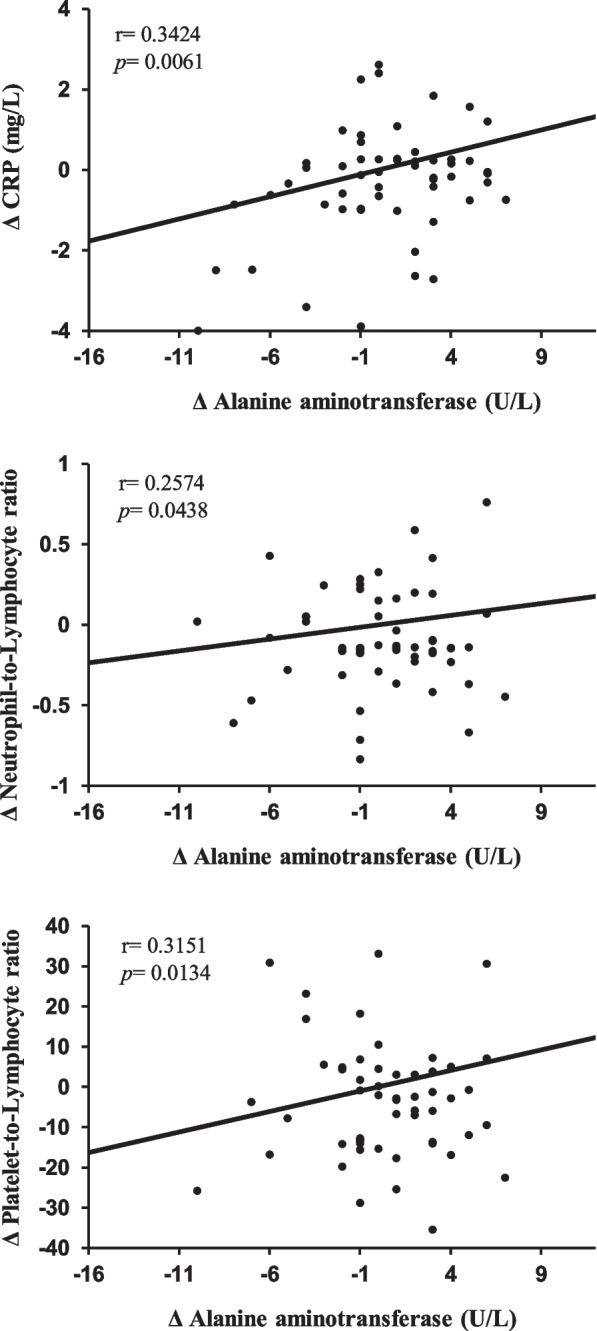
Serum Δ ALT concentrations as a function of Δ PLR, Δ NLR, and Δ CRP. Legend. Changes (Δ) in alanine aminotransferase, platelet-to-lymphocyte ratio, neutrophil-to-lymphocyte ratio, and C-reactive protein levels are expressed as values after nine months of treatment minus the baseline values

We analysed changes in the overall group of children with obesity (both with and without significant changes in their SDS-BMI; *n* = 63) with respect to the baseline. Single linear correlation showed positive associations between ALT level changes (Table 3) and changes in the SDS-BMI, WC, leptin (Fig. 2), insulin, HOMA-IR, and inflammation biomarkers (Fig. 3). Changes in serum BChE levels positively correlated with anthropometric measurements, IR, and inflammation biomarkers (Table 3).

**Table 3:** Single correlation coefficients (*r*) between changes in liver enzymes and different variables

	Δ ALT		Δ AST		Δ BChE		Δ ALT/AST	
	*r*	*p*	*r*	*p*	*r*	*p*	*r*	*p*
Δ BMI	0.3054	0.0149	0.2577	0.0435	0.5404	< 0.0001	0.2705	0.0331
Δ SDS-BMI	0.2691	0.0339	0.1669	0.1910	0.5150	< 0.0001	0.2564	0.0456
Δ WC	0.3436	0.0058	0.2869	0.0226	0.4607	0.0001	0.2638	0.0372
Δ Glucose	0.0270	0.8336	-0.1148	0.3703	-0.1881	0.1398	0.0618	0.6306
Δ Insulin	0.2602	0.0381	0.1243	0.3316	0.3036	0.0156	0.0779	0.5734
Δ HOMA-IR	0.2710	0.0329	0.1455	0.2550	0.2762	0.0318	0.0292	0.8200
Δ SBP	-0.2256	0.0731	-0.2278	0.0742	0.1192	0.3522	-0.1052	0.4117
Δ DBP	-0.2326	0.0628	-0.1702	0.1822	0.0543	0.6720	-0.2414	0.0572
Δ Total cholesterol	-0.0119	0.9261	0.1125	0.3801	0.1682	0.1876	-0.0783	0.5421
Δ Triglycerides	0.0623	0.6274	-0.0719	0.5755	0.4584	0.0002	0.0813	0.5266
Δ HDL cholesterol	-0.0290	0.8212	-0.0167	0.8966	-0.0116	0.9282	0.0255	0.8428
Δ CRP	0.3424	0.0061	0.2815	0.0264	0.3181	0.0115	0.2677	0.0354
Δ IL-6	0.2697	0.0337	0.2839	0.0242	0.0249	0.8462	0.1718	0.1818
Δ NLR	0.2574	0.0438	0.2348	0.0650	0.2702	0.0334	0.1540	0.2318
Δ PLR	0.3151	0.0134	0.2684	0.0351	0.2672	0.0364	0.2679	0.0353
Δ sICAM-1	0.0244	0.8497	0.1150	0.3693	0.0931	0.4678	-0.0135	0.9166
Δ Leptin	0.3264	0.0096	0.3039	0.0167	0.5291	< 0.0001	0.3677	0.0031

Results after treatment for nine months in the children with obesity group (*n* = 63). Changes (Δ) in different variables are expressed as values after 9 months of treatment minus baseline values

*ALT* Alanine aminotransferase, *AST* Aspartate aminotransferase, *BChE* Butyryl cholinesterase, *BMI* Body mass index, *WC* Waist circumference, *HOMA-IR* Homeostasis model assessment for insulin resistance, *SBP* Systolic blood pressure, *DBP* Diastolic blood pressure, *CRP* C-reactive protein, *IL-6* Interleukin-6, *NLR* Neutrophil-to-lymphocyte ratio, *PLR* Platelet-to-lymphocyte ratio, *sIACM-1* Soluble intercellular adhesion molecule-1

Using age and sex-corrected multivariate regression analysis for the children with obesity, changes in the SDS-BMI (*P* partial = 0.0294), WC (*P* partial = 0.0076), leptin (*P* partial = 0.0211), HOMA-IR (*P* partial = 0.0283), and inflammation biomarkers—IL-6 (*P* partial = 0.0090), CRP (*P* partial = 0.0065), and PLR (*P* partial = 0.0067)—were independent predictive factors for changes in ALT levels.

A multivariate regression analysis adjusted for SDS-BMI showed that changes in HOMA-IR (*P* partial = 0.0484) and the following inflammation biomarkers [CRP (*P* partial = 0.0260), NLR (*P* partial = 0.0492), and PLR (*P* partial = 0.0251)] were independent predictive factors for changes in ALT levels. However, changes in IL-6 (*P* partial = 0.0912) and leptin (*P* partial = 0.0526) levels were not predictive factors, even though they came close to reaching statistically significant levels.

For the serum BChE, age and sex-corrected levels of CRP (*P* partial = 0.0460), NLR (*P* partial 0.0425), PLR (*P* partial = 0.0395), anthropometric measurements (SDS-BMI* P* partial < 0.0001 and WC *P* partial = 0.0076), and leptin (*P* partial < 0.0001) were independent predictive factors.

## Discussion

In this work, we exclusively studied prepubertal children. The group of children with obesity who decreased their SDS-BMI after 9 months presented a decrease in the levels of liver enzymes, leptin, markers of IR, inflammation and endothelial dysfunction, and variables associated with MetS. Moreover, these changes in liver enzymes levels were associated with altered IR, inflammatory biomarkers, and leptin.

MetS can start in children with obesity at very young ages [1], even before puberty [25]. Furthermore, the presentation of this syndrome in children is associated with a high risk of diabetes and atherosclerotic cardiovascular disease during adulthood [26].

Moreover, NAFLD is associated with obesity and MetS [7, 27] and its prevalence increases in line with that of MetS and the components of MetS, particularly obesity [28]. Indeed, some authors consider these to be hepatic manifestations of metabolic syndrome [6]. NAFLD has now become the most frequent cause of chronic liver disease, both in children and adults [29]. Exercise interventions and lifestyle changes reduce fat mass [30] and liver fat, as well as the prevalence of NAFLD in children and adolescents, and have been recommended in the treatment of paediatric obesity [30, 31] and hepatic steatosis [16].

### Liver enzymes, insulin resistance, and parameters related to metabolic syndrome

The prevalence of IR is correlated with the BMI category and weight reduction is an important measure in the fight against IR in children [32]. Indeed, children with increased ALT levels (as surrogate markers for NAFLD), showed higher prevalences of prediabetes and type-2 diabetes mellitus compared to those with normal ALT levels [33].

In this present work, children with obesity who decreased their SDS-BMI had lower levels of ALT and IR markers. Changes (the baseline levels subtracted from the results at 9 months of treatment) in ALT and BChE levels were associated with alterations in the same direction in BMI, insulin, and HOMA-IR values. Moreover, when corrected for age, sex and SDS-BMI, changes in ALT levels were still associated with altered HOMA-IR. NAFLD is closely related to IR and hyperinsulinemia, which favours an increase in the levels of free fatty acids, TG, and the onset of hepatic steatosis [33]. Indeed, in obese children older than those studied in this current work, liver enzyme levels were significantly associated with a reduction in insulin sensitivity [34].

We found that there was a decrease in HOMA-IR and improvement in the lipid profiles of children with obesity who decreased their SDS-BMI. Stratification of the severity of obesity, using SDS-BMI, was effective in estimating cardiometabolic risk [35]. When classifying the group of children with obesity according to ALT, the upper tercile showed the highest values for insulin, HOMA-IR, TG. The metabolic profile, especially HOMA-IR, was altered, even in young children with obesity [35]. Hepatic steatosis was considered an important factor in the early pathogenesis of IR and type 2 diabetes in young people [36].

Blood pressure figures were also lower in children who decreased their SDS-BMI, although this factor was not correlated with liver enzyme values. Although some authors have suggested that ALT is a potential indicator of hypertension [37], according to others, NAFLD was not associated with blood pressure after adjusting for the degree of obesity [38, 39]. In line with this suggestion, children with obesity and with NAFLD have a higher risk of hypertension compared to those without NAFLD [40]. Thus, at prepubertal ages there is an association between liver enzyme levels, anthropometric measurements, insulin, and HOMA-IR in children with obesity.

### Liver enzymes, leptin, and inflammation biomarkers

Adipose tissue plays an important role in the pathogenesis of NAFLD; it interacts with the liver and releases a series of adipokines involved in processes such as inflammation [12], insulin sensitivity, and NAFLD. Elevated levels of leptin have been associated with NAFLD and its serum concentration correlates with the severity of NAFLD [13]. Insulin resistance triggers the synthesis of several proinflammatory mediators and prodiabetogenic hepatokines that can promote the development of type 2 diabetes. Thus, the prevalence of prediabetes and MetS increases significantly in line with liver fat content [41].

We found a significant association between treatment-induced changes in liver enzyme levels (particularly ALT) and inflammation markers, IR, and leptin in children with obesity aged 6–9 years. When divided according to the ALT terciles, the upper tercile showed the highest levels of biomarkers for inflammation and leptin. Leptin increases IR and promotes inflammatory and fibrogenic pathways in the liver [42]. Furthermore, serum CRP levels are predictive of NAFLD and have been related to the presence and severity of liver fibrosis [43].

Taken together, this evidence suggests that, from an early age, adipose tissue may be involved in the onset of the metabolic disorders that accompany obesity and its complications such as MetS and NAFLD. Our results support this idea and indicate that the disorders that accompany obesity and NAFLD can benefit from the standard treatment, even before puberty. Further studies will be needed to assess the development of childhood overweight/obesity problems and to estimate the effectiveness of lifestyle changes with the aim of developing personalized treatment procedures [44].

Early diagnosis and therapy during disease stages in which hepatic steatosis may still be reversible are essential to preventing further progression. Lifestyle changes produce significant improvements in BMI, ALT levels, and hepatic steatosis in children and adolescents with NAFLD [45]. Moreover, non-invasive non-alcoholic fatty liver markers (ALT and steatosis) tend to improve upon combined lifestyle and exercise improvements [46, 47]. Nonetheless, conducting treatment trials for NAFLD in children remains challenging because of the lack of non-invasive biomarkers and insufficient knowledge of the natural history of the disease.

However, lowering ALT may be an acceptable surrogate in NAFLD to assess response to treatment, particularly in the early stages [16]. There is some evidence to support its use in paediatric clinical trials [48]. Some studies also describe ALT as a value whose fluctuation, even within normal value ranges, indicates a risk of cardiovascular diseases [49].

Not performing certain imaging studies may have been a limitation of this study. Our objective was to analyze the biochemical parameters related to obesity and NAFLD that are low-cost and are easy to apply in clinical practice. There are several reasons why this age was chosen. One of them is that we exclusively analyzed prepubescent children, thus eliminating the possible differences attributable to the onset of puberty. The prevalence of obesity is worrying at this age and childhood is a stage in which we can still intervene to get children to acquire good lifestyle habits that make it possible to prevent the onset of obesity.

Thus, these variables could add valuable information to the evaluations of children with obesity and NAFLD. Notwithstanding, more studies will be required to determine the optimal age for the detection of NAFLD.

## Conclusions

Our results showed that a decrease in ALT levels after the standard treatment for 9 months was associated with favourable changes in IR markers (HOMA-IR) and inflammation (IL-6, CRP, NLR, and PLR). Lower liver enzymes improve these variables.

By classifying children with obesity according to their ALT expression terciles, we found the highest values of these variables were in the upper tercile of ALT.

Liver enzymes, parameters related to inflammation and adipokines could add information to the evaluation and monitoring in children with obesity.

## Data Availability

The data used to support the findings of this study are available from the corresponding author upon request.

## Abbreviations

MetS: Metabolic syndrome
NAFLD: Non-alcoholic fatty liver disease
IR: Insulin resistance
ALT: Alanine aminotransferase
BChE: Butyryl cholinesterase
BMI: Body mass index
AST: Aspartate aminotransferase
sICAM-1: Soluble intercellular adhesion molecule-1
CRP: C-reactive protein
IL-6: Interleukin- 6
TG: Triglycerides
HDL-c: High-density lipoprotein cholesterol
HOMA-IR: Homeostasis model assessment for IR
SEM: Standard error mean
